# Entosis: the core mechanism and crosstalk with other cell death programs

**DOI:** 10.1038/s12276-024-01227-w

**Published:** 2024-04-02

**Authors:** Sunghoon Kim, Donghyuk Lee, Sung Eun Kim, Michael Overholtzer

**Affiliations:** 1 Department of Biosystems and Biomedical Sciences, College of Health Sciences, Korea University, Seoul, Republic of Korea; 2 Department of Integrated Biomedical and Life Sciences, College of Health Sciences, Korea University, Seoul, Republic of Korea; 3 L-HOPE Program for Community-Based Total Learning Health Systems, Seoul, Republic of Korea; 4 Department of Pharmacology and Brain Korea 21 Project for Medical Science, Yonsei University College of Medicine, Seoul, Republic of Korea; 5 Cell Biology Program, Sloan Kettering Institute for Cancer Research, New York, NY USA; 6 Louis V. Gerstner, Jr. Graduate School of Biomedical Sciences, Memorial Sloan Kettering Cancer Center, New York, NY USA; 7 BCMB Allied Program, Weill Cornell Medical College, New York, NY USA

**Keywords:** Tumour heterogeneity, Entosis

## Abstract

Cell death pathways play critical roles in organism development and homeostasis as well as in the pathogenesis of various diseases. While studies over the last decade have elucidated numerous different forms of cell death that can eliminate cells in various contexts, how certain mechanisms impact physiology is still not well understood. Moreover, recent studies have shown that multiple forms cell death can occur in a cell population, with different forms of death eliminating individual cells. Here, we aim to describe the known molecular mechanisms of entosis, a non-apoptotic cell engulfment process, and discuss signaling mechanisms that control its induction as well as its possible crosstalk with other cell death mechanisms.

## New insights into cell death mixtures: entosis mechanisms unveiled

Cell death, a complex process, can happen through various mechanisms, including entosis. Entosis, first identified as a non-apoptotic cell death program, involves one cell engulfing another, causing the death of the internalized cell. However, the exact molecular mechanisms and factors controlling entosis are unclear. In this study, the authors review recent findings uncovering the molecular regulation of entosis and dicuss its potential implications in cancer. They discuss how entosis is controlled by a network of regulations involving cell adhesion, the RhoA–ROCK signaling pathway, and actomyosin contraction. Interestingly, entosis can occur alongside other cell death forms in populations, suggesting it may influence how cell groups respond to stress. The study concludes that entosis may contribute to aggressive cancers’ development by favoring cells with low tension setpoints, promoting the creation of aneuploid cell lineages, and supporting cell lineages with large scavenged nutrient sources.

This summary was initially drafted using artificial intelligence, then revised and fact-checked by the author.

## Introduction

Cell death mechanisms can be categorized according to the distinct molecular regulatory mechanisms that control their execution. Entosis was first identified as a non-apoptotic cell death program induced by live-cell engulfment^1^. The mechanisms that control entosis are well established and include calcium-dependent adhesion (cadherin)- and Ras homologous (Rho)-associated kinase (ROCK)-mediated cell-in-cell formation followed by non-canonical autophagy- and lysosome-mediated entotic cell death. Additional regulatory mechanisms of entosis involve the activity of AMP-activated protein kinase (AMPK), the stress kinases c-Jun N-terminal kinase (JNK) and p38, the oncogenes Kirsten rat sarcoma 2 viral oncogene homolog (KRas) and cellular Myc (c-Myc), the p53 tumor suppressor protein, and other proteins. Here we discuss the core molecular mechanism of entosis and consider how it is impacted by these additional layers of regulation, and we examine the roles of this complex process in cancer and its crosstalk with other cell death mechanisms. For clarity, we focus on the mechanism of cell-in-cell formation in entosis and not on downstream entotic cell death, which has been reviewed elsewhere^2–7^.

## Entosis: cell adhesion-based cell-in-cell formation in cancer

**Fig. 1:**
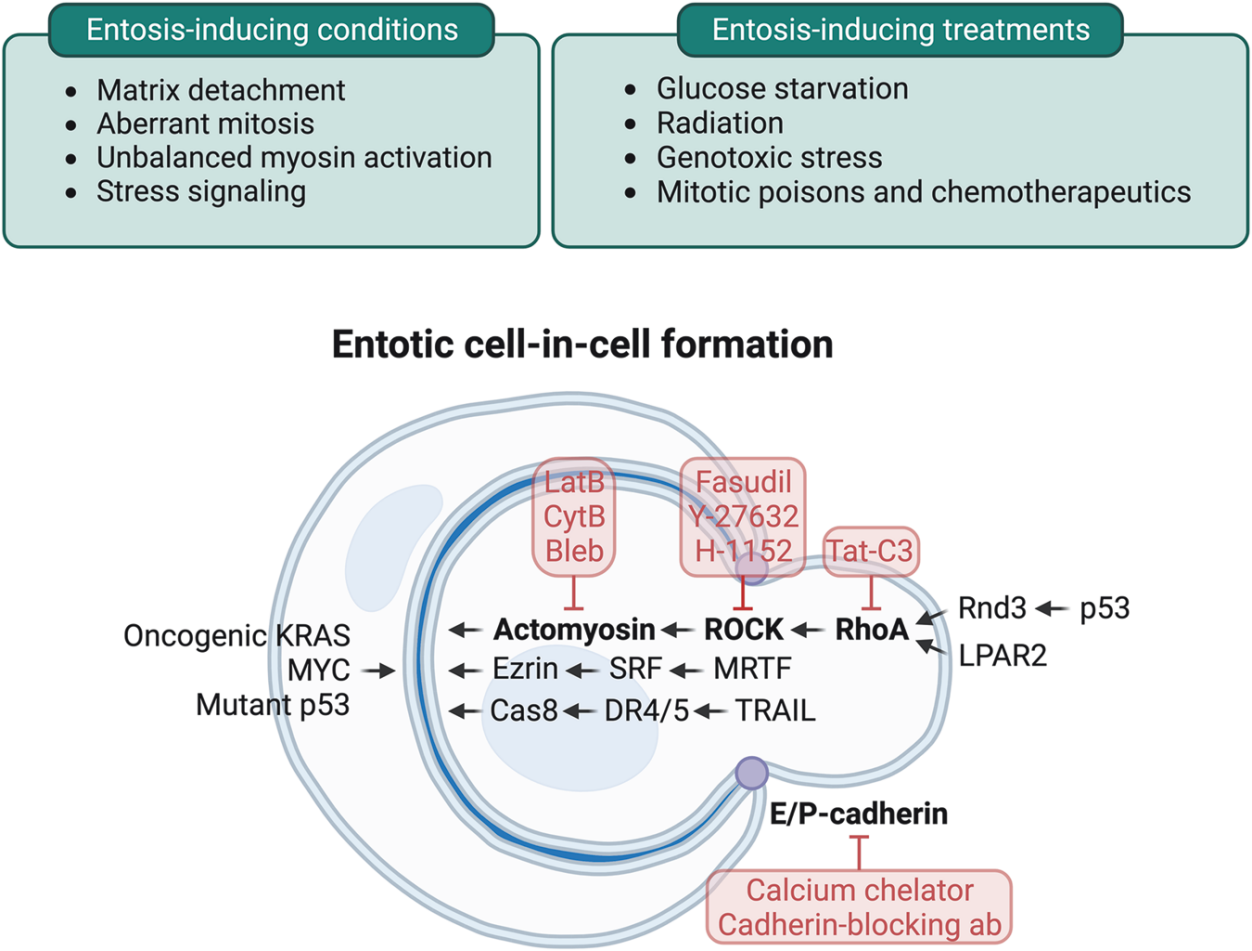
The core entosis mechanism and its regulatory pathways. Top: Conditions and treatments that lead to the induction of entosis through regulation of the core mechanism. Bottom: The core mechanism of entotic cell-in-cell structure formation consists of cell‒cell adhesion and actomyosin contraction, depicted in bold. Between neighboring cells with adherens junctions formed through cadherin molecules, the differential mechanical deformability imparted by RhoA–ROCK signaling-mediated actomyosin contraction serves as the driving force for the internalization of the engulfed, or “loser”, cell into the engulfing, or “winner”, cell. Inhibitors blocking each component of the core mechanism are depicted in red. The core entosis mechanism can be regulated by various pathways induced by oncogenes, tumor suppressor genes, or stress signaling.

Entosis was first characterized as a cell death mechanism induced by the loss of cell adhesion to matrix, occurring through the formation of “cell-in-cell” structures between neighboring epithelial cells, a process named based on the appearance of whole cells that become internalized inside of other cells^1^. While several mechanisms can induce cell-in-cell formation and have been reviewed extensively elsewhere^8–11^, here we focus specifically on entosis. Entosis requires the formation of cell adhesions between the engulfing, or “host”, cell and the cell that becomes internalized, through adherens junctions formed by cadherin molecules (Fig. 1). Accordingly, entosis is inhibited by cadherin-blocking antibodies or by the chelation of calcium, which is required for cadherin-mediated adhesion^1^. The expression of epithelial-type cadherins, including epithelial cadherin (E-cadherin) and placental cadherin (P-cadherin), as well as α-catenin, the linker between cadherins and the cytoskeleton, in cells lacking these proteins can restore the ability of cells to undergo this process^12,13^.

Entosis is observed in various cancers, including numerous carcinomas, potentially owing to their epithelial origin and thus expression of epithelial cadherins^9^. While in some cases, acute induction of entosis can inhibit transformed growth due to the death of the internalized cells^12,13^, on balance, this process occurs more frequently in aggressive cancers and is thought to promote disease progression^14^. Entosis may promote cancer progression due to its ability to elicit competition between individual cells in a manner similar to “cell competition” first described in the context of normal tissue development^15^. Cells with more aggressive characteristics, or “winner” cells, can internalize and kill those with less aggressive characteristics, called “loser” cells. Winner cells also scavenge nutrients from internalized loser cells^16^ and propagate aneuploid lineages due to the disruption of cytokinesis that can result from cell-in-cell formation^17^. Thus, while entosis leads to cell death and its acute induction can slow transformed growth, in the long term, this process may contribute to the development of aggressive cancers.

## The core entosis mechanism: RhoA activity and actomyosin contractility

Adherens junctions coordinate cell adhesion, cell shape changes, and cell movement by linking to the actin cytoskeleton, and accordingly, cytoskeletal dynamics play an important role in regulating entosis. Treatment with the actin polymerization inhibitors latrunculin B and cytochalasin B or with blebbistatin, an inhibitor of myosin II, block this form of cell engulfment^1,18^. The Rho-ROCK pathway, a key regulator of actomyosin contraction, is also required for entosis, as inhibition of Rho GTPases by treatment with Tat-C3 or ROCK inhibition by treatment with Y-27632 or H-1152 blocks the formation of cell-in-cell structures through this mechanism^1^. Pre-treatment of cells with Tat-C3 or ROCK I/II-targeting siRNAs can prevent cells from internalizing into their neighboring cells while not affecting their ability to act as hosts, demonstrating that, surprisingly, RhoA and ROCK act specifically within internalizing cells to promote this process^1^. RhoA activity, as measured by a FRET-based biosensor, and ROCK I/II expression are increased within internalizing cells, localized to cortical regions opposite cell adhesions, consistent with the polarized activity of RhoA and ROCK as the main drivers of this process^12^. Similar to RhoA activity, the expression levels of myosin heavy chain (MHC) IIA and IIB and the level of myosin light chain 2 phosphorylated at serine 19 (pMLC2), a readout of contractile myosin downstream of ROCK, are higher within internalizing cells and localized to the cortex opposite cell junctions^12^. Overall, these findings support a model in which differential mechanical deformability between neighboring cells provides a key driving force to promote this form of cell engulfment^15^.

### Regulation of RhoA and actomyosin

The recruitment of the RhoA inhibitory protein p190 Rho GTPase activating protein (GAP) to cell junctions may be at least partially responsible for polarizing myosin contractility during entosis, as its depletion inhibits entosis induction under matrix detached conditions and redistributes contractile myosin to a more uniform pattern around the cell cortex^12^. Overexpression of p190 RhoGAP or inhibition of RhoA by overexpression of dominant negative RhoA-N19 is sufficient to prevent cells from internalizing into their neighboring cells, whereas overexpression of RhoA, ROCK I, or ROCKII has the opposite effect and can drive cell internalization, generating loser cells^15^. These findings suggest that difference in the setpoint of RhoA–ROCK signaling between neighboring cells is a key determinant of entosis induction and that loser cells are characterized by increased actomyosin contractility, which becomes polarized during the formation of cell‒cell adhesions.

Consistent with this model, Purvanov et al. further reported that the G protein-coupled receptor lysophosphatidic acid receptor 2 (LPAR2) promoted entosis by activating RhoA through the heterotrimer Gα12/13 and through localization of the RhoA activator protein PDZ–Rho guanine nucleotide exchange factor (GEF) to the cortex localized opposite from cell adhesions^19^. Thus, collectively, the actions of p190 RhoGAP localized at cell adhesions and PDZ–RhoGEF localized away from cell adhesions contribute to polarized actomyosin contractility and drive entosis by promoting the internalization of more contractile cells into less contractile neighboring cells. The idea that internalizing cells are stiffer or less deformable than host cells was confirmed by biophysical measurements of entotic structures performed by micropipette aspiration and by computational modeling, which predicted the sufficiency of a mechanical differential to promote this cell adhesion-dependent process^15^. Notably, the described localization of contractile actomyosin and RhoA activity during entosis closely resemble those identified during the formation of normal cell adhesions between adherent cells, suggesting that entosis results from normal cell adhesion-related processes that become unbalanced^20^.

### Additional regulation of the core mechanism

While adherens junctions, RhoA–ROCK signaling and actomyosin contraction form the central regulatory network that controls entosis, other key regulatory components that affect cell adhesion or cytoskeletal dynamics may also influence this process. For example, a structure named the “mechanical ring” was identified; this structure is characterized by the presence of the mechanosensitive cell adhesion–actin cytoskeleton linker protein vinculin, which is localized in a large ring-like structure at the interface of internalizing and host cells, between adherens junctions and accumulated actomyosin. This structure may link the adherens junction protein α-catenin to the actin cytoskeleton through a mechanosensitive interface as junctions are remodeled during entosis^21^. Depletion of vinculin indeed leads to actomyosin depolarization and the inhibition of entotic cell-in-cell structure formation.

Another actin-binding protein, ezrin, a member of the ezrin–radixin–moesin (ERM) family of cytoskeleton–membrane linker proteins, is also a direct regulator of this process. Hinojosa et al. showed that ezrin is important for entosis induction and is regulated by the myocardin-related transcription factor (MRTF)–serum response factor (SRF) pathway through membrane blebbing, which is observed at the internalizing cell cortex but not in the host cell^19^. Transcriptional upregulation of ezrin in response to blebbing occurs at the cortex, opposite the cell–cell adhesion interface; this upregulation stabilizes cortical bleb dynamics and drives entotic cell internalization, demonstrating further coordination between the regulation of actin dynamics and entosis. These studies also showed that the process of entosis is regulated by transcription through a mechanical feedforward loop.

Additional cytoskeletal or cortical regulators of entosis that affect cell tension and/or RhoA–ROCK pathway activity include interactions with microtubules^18,22^, cell polarity proteins^23^, calcium signaling and interaction with Septins and myosin light chain kinase (MLCK)^24^, myosin light chain phosphatase (MYPT1)^25^, membrane composition and cholesterol^26^, and small GTPase proteins, including cell division cycle 42 (Cdc42)^27,28^ and, intriguingly, ras-related C3 botulinum toxin substrate 1 (Rac1)^15,25^. Rac1 is a key driver of phagocytosis, during which it regulates the remodeling of the actin cytoskeleton into dendritic networks within engulfing cells, such as macrophages. During entosis, Rac1 may regulate cell internalization by modulating RhoA activity and tension within internalizing cells. Loss of Rac1 expression leads to upregulation of RhoA and increased actomyosin contractility, which promotes the internalization of cells into neighboring cells. Intriguingly, the knockdown of ROCK I/II turns Rac1-depleted cells into hosts, demonstrating that entotic cell engulfment, unlike phagocytosis, is primarily a Rac1-independent process, although Rac1 can still influence entosis by affecting the relative setpoint of RhoA–ROCK activity between neighboring cells^15^. The small GTPase and potent oncogene KRas may influence entosis through a similar mechanism, where cells expressing mutant KRas act as hosts or winner cells due to the upregulation of Rac1 and concomitant downregulation of RhoA and decrease in mechanical tension^15^.

### Modes of entosis induction affecting the core mechanism

Entosis was first identified in mammary epithelial cells, breast cancer cells, and other cell types cultured in suspension^1^. Since its discovery, entosis has been observed in numerous additional cellular contexts, including adherent cell populations, raising the question of how this process is induced. As the primary regulation of entosis involves force imbalances between neighboring cells, conditions that induce population-scale heterogeneities in cellular mechanics may often be drivers of this mechanism.

Under adherent conditions, entosis was first shown to occur in cells overexpressing or depleted of cell polarity proteins (lethal (1/2) giant larvae (Lgl1/2) and cell polarity protein partitioning defective 3 (Par3)), which led to robust myosin activation. In mixed cultures, entosis resulted in the internalization of contractile Lgl1/2-depleted or Par3-overexpressing cells into control neighboring cells with lower setpoints of myosin activation, demonstrating that genetic disruptions to the normal regulation of cell mechanics can generate heterogeneities that may be sufficient to drive this process^23^. Perhaps consistent with this model, Schwietzer et al. showed that loss of the cell adhesion receptor junctional adhesion molecule A (JAM-A), a regulator of tight junctions, in breast cancer cells increased the entosis in matrix-adherent populations but not in cells in suspension^29^. Loss of JAM-A suppressed contact inhibition of locomotion (CIL), leading to a cell state in which motility was maintained even upon cell contact, a scenario where entosis might be likely to occur as cell-in-cell formation is driven even after the establishment of cell‒cell adhesions. In this context, motile cells with JAM-A depletion displayed increased cellular stiffness and were internalized into control cells, again demonstrating that alterations in mechanical tension in adherent cell populations can induce entosis by driving imbalances between neighboring cells.

On the other hand, Durgan et al. showed that entosis can occur in adherent cells as a result of aberrant mitotic events^27^. Depletion or inhibition of the small GTPase Cdc42 or repressor/activator protein 1 (Rap1), was found to induce entosis by altering the mechanics of mitosis, characterized by increased cell rounding, elevated RhoA activity and more prominent cortical actin at metaphase. Notably, mitotic entry was required for entosis to occur in adherent cultures but not in suspended cells, and mitotic entosis could not be mimicked by placing suspended cells on top of adherent cells. These findings demonstrated an alternative pathway of entosis induction in adherent populations that is linked to altered mitotic events. The authors observed numerous cancer cell lines exhibiting “mitosis-induced entosis” in unperturbed cultures, as well as entotic structures involving mitotic cells in human tumor samples, suggesting that mitotic rounding and the associated increase in cell contractility may be a common entosis-inducing mechanism. Intriguingly, cancer cells treated with inhibitors of the mitotic kinase Plk1 or the chemotherapeutic drugs abiraterone or Taxol also underwent entosis due to mitotic disruption, suggesting that this mechanism could have clinical significance in cancer therapy^30^.

Adding to this model, Liang et al. reported that entosis occurring in adherent cultures of mammary epithelial cells or breast cancer cells also involved aberrant mitotic events, linked specifically to attempted division of aneuploid cells^31^. Similar to cells with Cdc42 inhibition, aneuploid cells exhibited a prolonged metaphase, resulting in sustained cell rounding and increases in RhoA activity and contractile myosin, demonstrating again that aberrant mitotic events are sufficient to trigger entosis. Intriguingly, the authors discovered that activation of the p53 tumor suppressor protein within internalizing cells, resulting from an observable increase in DNA damage, was required to initiate entosis in this context. Moreover, the p53 effector small GTPase protein rho-related GTP binding protein RhoE (Rnd3) was found to be localized to cell‒cell adhesions and to contribute to polarizing actomyosin toward the rear cortex of internalizing cells. Similar to the effects of p190 RhoGAP inhibition discussed above, Rnd3 depletion redistributed contractile myosin from a polarized to a more uniform cortical distribution, with increased localization at cell‒cell adhesions^31^. Taken together, these findings link aberrant mitotic events to activation of the core mechanism of entosis and demonstrate that entosis can eliminate cells identified as defective by their inability to divide normally, revealed through a prolonged metaphase and changes in cell shape and actomyosin tension.

## Signaling pathways affecting the core entosis mechanism

### Stress signaling through AMPK and JNK/p38

While enforced mechanical imbalances and aberrant mitotic events can initiate entosis, recent findings have revealed that stress signaling can also impose heterogeneities that lead to entosis induction. First, Hamann et al. showed that nutrient starvation in the form of glucose withdrawal is a significant inducer of entosis via signaling through AMP-activated protein kinase (AMPK), which is activated by low-energy states through the direct binding of AMP and ADP^32^. In starved cell populations, AMPK was activated to a higher extent within entotic internalizing cells than within host cells, leading to the elimination of cells with high levels of stress signaling. Consistent with the activation of the core entosis mechanism, AMPK was required for the generation of stiffer cells within the population in response to glucose withdrawal, and its activation correlated with the phosphorylation of MLC2 at Ser19, a ROCK target site, which mediates contractility^32^. These findings showed that heterogeneities in stress signaling in a cell population can engage core entosis regulation, and they implicated bioenergetic collapse as another important entosis inducer. Recently, additional studies have shown that defective glycosylation in glucose-starved cells is also linked to entosis induction in response to starvation^33^.

In addition to the abovementioned influence of AMPK, another study by Chen et al. revealed that signaling through the stress kinases JNK and p38 can induce entosis in cell populations exposed to ultraviolet (UV) radiation^34^. Here, stress kinase signaling was induced in all cells in the population after UV exposure, but over time, some cells exhibited decreased signaling, presumably due to recovery from stress, and these cells became entotic hosts for neighboring cells with persistent high levels of stress signaling. While this study did not identify how JNK and p38 signaling control loser cell behavior, it is conceivable that high levels of stress signaling, which are linked to mechanical tension^35^, could promote mechanical differentials between neighboring cells.

### Oncogenes and tumor suppressors

The frequent appearance of entotic structures in cancer tissues prompts additional consideration of genetic backgrounds related to known oncogenes or tumor suppressors that may influence the formation of these structures. Cancer cells have been demonstrated to generally act as winners during entosis when co-cultured with noncancer cells^15^, suggesting that cancer-associated mutations may provide cells with competitive advantages during entosis, an effect potentially linked to the known increase in deformability of cancer cells compared to noncancer cells^36^.

The classic and potent oncogenes *KRas* and *c-Myc* have been implicated in regulating entosis in a manner where their activation may confer winner status. The expression of mutant *KRas*, as discussed above, can increase mechanical deformability and lower the level of pMLC2 Ser19 in a Rac1-dependent manner, suggesting that KRas influences entosis primarily through modulation of its core mechanism^15^. This effect of mutant *KRas* in promoting winner cell behavior has been shown both in the context of entosis induced in suspended cultures and in mixed monolayer cultures, where cells with *KRas* mutations and loss of expression of the cell polarity protein Scribble were shown to act as winners and to eliminate neighboring *KRas* wild-type cells through entosis^37^. *KRas* amplification has been shown to correlate with the presence of entotic structures in pancreatic carcinoma, suggesting a potential link between KRas signaling and the induction of entosis in human cancers^38^.

*c-Myc* is also linked to entosis induction in pancreatic cancer tissues, where it has been found to exhibit greater amplification in entotic host cells than in internalized cells^14^. However, the mechanism underlying the influence of *c-Myc* on entosis induction is not well understood. *c-Myc* expression was shown to confer winner status—intriguingly, in a JNK-dependent manner—in a model of cannibalistic engulfment in cultured breast cancer cells, but whether entosis was the underlying mechanism in this system was not clearly defined. *c-Myc* is also a key oncogene that drives competitive advantages within developing tissues, promoting a “supercompetitor” status in cells that cause the death of their wild-type neighbors, suggesting further potential similarities between entosis and mechanisms of cell competition.

The p53 tumor suppressor protein is also a regulator of entosis. As discussed above, the activation of p53 in aberrant mitotic cells, which occurs downstream of DNA damage, can induce entosis via exerting effects within internalizing cells that are cleared through this mechanism. It has also been shown that heterogeneous cancer cell populations, defined by either mutation or deletion of *p53* in individual cells, exhibit increased entotic activity. Surprisingly, cells with mutant *p53* preferentially exhibited winner behavior over cells with *p53* deletion. Here, a possible link between mitosis and the induction of entosis was also noted, and epidermal growth factor receptor (EGFR) and alpha 5 integrin were shown to be required for entosis induction, identifying important new regulators of this process that may be specific to adherent conditions. The authors further discovered that *p53* mutant cells exhibited enhanced survival as entotic hosts; this was linked to both the occurrence of replication stress in host cells and to the disruption of cytokinesis, an effect of entosis that can be lethal to host cells^17^. These findings revealed further connections between p53 dysfunction and the induction and dynamics of entosis.

In addition to *p53*, one other tumor suppressor gene, cyclin-dependent kinase inhibitor 2A (*CDKN2A)*, has also been shown to regulate entosis. Surprisingly, the expression of either p16INK4a or p19ARF, two tumor suppressor proteins encoded by *CDKN2A* that function in cell cycle arrest and p53-responsive pathways, can suppress the induction of entosis in suspension cultures^39^. Overexpression of these proteins was shown to reduce myosin contractility and to generate winner cells when co-cultured with control cells in suspension. Intriguingly, the surface expression of E-cadherin was also reduced in these cells, suggesting that relatively low levels of E-cadherin could be sufficient to mediate entosis or, alternatively, that additional cadherins may compensate for E-cadherin loss in this context. In human breast cancer specimens, the frequency of entotic structures was increased in tumor regions with low levels of staining for p16INK4a, suggesting, overall, that the expression of tumor suppressor proteins encoded by *CDKN2A* reduces the frequency of entosis in cancer^39^.

## Crosstalk between entosis and other forms of cell death

The core regulatory framework of entosis—involving cell‒cell adhesion, RhoA–ROCK signaling, and actomyosin—is central to many aspects of epithelial cell biology; thus, many potential avenues of crosstalk with other forms of cell death may exist. For example, alterations in the actin cytoskeleton, involving either increased or decreased actin polymerization or myosin tension, can alter sensitivity to apoptosis as well as entosis^40^. Recent studies have also revealed mechanical inputs controlling apoptosis during cell competition, both in *Drosophila*^41,42^ and in mammalian cells, where increased crowding of cells triggers activation of ROCK and apoptosis through signaling mediated by the p38 stress kinase and p53^43^, which are also regulators of entosis and other forms of cell death. As mechanical tension setpoints are regulators of multiple forms of cell death, including even cytotoxic T-cell-mediated killing^44^, and increased tension can be sufficient to induce cell death^45^, further consideration of this fundamental cell property as a mechanistic connection between entosis and other forms of cell death seems warranted.

Changes in cell adhesion-based signaling or gene expression can also affect multiple death processes in complex ways. E-cadherin expression, which is required for entosis in suspension culture, can promote either resistance^46^ or sensitivity^47^ to a specific mode of apoptosis referred to as “anoikis”^48^. The expression of E-cadherin may also regulate entosis in complex ways. While E-cadherin expression is required for this process in some contexts^1^, recent reports have linked decreased expression of E-cadherin in cancers to entosis induction, a relationship potentially associated with epithelial-to-mesenchymal transition (EMT)^49,50^. Liver cancer cells have also been shown to undergo entosis in an E-cadherin-independent manner, curiously, when Rnd3 or p190 RhoGAP are silenced^51^. Mechanistic studies linking entosis to loss of the small GTPase Cdc42, as discussed above, have also demonstrated that cells can undergo entosis when the maturation of E-cadherin-based junctions is disrupted, suggesting that immature adhesions, called “primordial junctions”, may be sufficient to mediate this process^27^. Moreover, decreased levels of surface E-cadherin expression are associated with winner status in cells with forced expression of the *CDKN2A*-encoded tumor suppressor proteins, as discussed above, again suggesting that while the expression of epithelial cadherins is required for entosis to occur in many contexts, they might also regulate this process in complex ways^39^. Recent studies have shown that cadherin-based signaling also determines sensitivity to additional forms of cell death, including necrotic cell death through the iron- and lipid peroxidation-dependent mechanism of ferroptosis. Here, junctional signaling through the Hippo–YAP pathway ultimately links the transcriptional regulation of iron uptake and membrane composition to relative ferroptosis sensitivity. In this context, adherens junctions reduce YAP activation and thereby promote resistance to cell death^52^.

While different signaling mechanisms affect individual forms of cell death^53^, death occurring in response to stress^32,34^ or infection^54^ in cell populations may manifest as mixed profile, where individual cells die via different mechanisms. Intriguingly, entosis and anoikis, as well as necrosis and cornification^55,56^, can occur in parallel as complex mixtures within cells cultured in suspension^1^. Similarly, while entosis is induced by glucose starvation or UV exposure, other forms of cell death, including apoptosis and necrosis, can also occur in parallel. For example, in UV-treated MCF-7 breast cancer cells, entosis was responsible for ~50% of the cell death events, while necrosis and apoptosis were responsible for ~30% and ~20%, respectively; in contrast, in BxPC-3 pancreatic cancer cells, entosis and necrosis were responsible for only a small percentage of cell death events, while apoptosis predominated at ~90%. Notably, treatment of BxPC-3 cells with the pan-caspase inhibitor z-VAD-fmk inhibited apoptosis but did not significantly reduce the overall death rate in the population, as the percentages of entotic and necrotic cell death were increased significantly^34^. Treatment of MCF-7 cells with the ROCK inhibitor Y-27632, on the other hand, inhibited entosis but led to a significant increase in necrosis to account for approximately 80% of cell death events, an effect also observed when entosis was inhibited in glucose-starved populations. Taken together, these findings demonstrate that complex mixtures of cell death modes can occur in cell populations in response to stress, and that inhibition of one mode may not rescue the population, as cells may die through alternative mechanisms.

Uncovering how mixed death modes are regulated—and how the fates of individual cells are determined—may shed new light on how cell populations respond to stress and how death occurs in clinical contexts, such as during cancer therapy, where mixed death modes may also occur. It is possible that common signaling nodes can be identified that control multiple forms of death execution. Numerous regulators of entosis that have been discussed herein, including p53, AMPK, and JNK/p38, are also central to the regulation of other forms of cell death, suggesting that these common regulators could function upstream in the induction of numerous different forms of cell death in populations. Treatment of colon cancer cells with the apoptosis-inducing factor TNF-related apoptosis-inducing ligand (TRAIL), a well-known inducer of apoptosis, was also recently shown to induce entosis; intriguingly, caspase-8 was shown to be required for this induction. The TRAIL-responsive pathway was also correlated with entosis induction in colorectal cancer specimens, suggesting a new mechanism of entosis induction in human cancers^57^. It is conceivable that caspase-8 is one example of a nucleation point for protein complexes that inform cell fate decisions involving a wide range of death modes, from apoptosis to necrosis and also entosis.

## Concluding remarks

Here we have discussed the mechanism of entosis and its regulation by numerous conditions and factors that have been found to control its induction. While entosis is clearly induced as a stress response, similar to and in parallel with other forms of cell death, it also has unique features, and its physiological significance remains poorly understood. Entosis is well-known to occur in cancers, but whether this process has functions in normal physiology remains an open question, although several reports have shown the occurrence of cell-in-cell formation that directly resembles entosis and is regulated by entotic factors occurring in normal development, including during embryo implantation in mice^58^, male gonadal development in *C. elegans*^59^, and germ cell differentiation^60^. Whether parallels between entosis and forms of cell competition that function to promote fitness in developing tissues also represent more direct mechanistic connections between these processes that have been suggested by culture models^15,37^, awaits further studies of competition mechanisms in tissue and whole organism levels.

In summary, entosis appears to select for relative setpoints of mechanical tension in cell populations by mediating the clearance of cells with high tension by those with lower tension. In noncancer tissues, this effect may eliminate dysfunctional cells and act in a tumor-suppressive manner, for example, through a clearly established function to eliminate cells exhibiting aberrant mitosis^27,31,61^ or defective polarity^23,61^, as well as through the observed effect of entosis on limiting transformed growth^1,62^. Tumor-suppressive activity has also been shown in a zebrafish model where entotic internalization was shown to limit the growth of epidermal hyperplasia^63^. In the long term, however, the ability of entosis to select for “fitness” in this manner likely promotes selection for oncogene-expressing cells and can thereby accelerate the development of aggressive cancers, a concept supported by numerous studies that have linked the presence of entotic cell structures to high-grade cancers with poor prognosis^8^. Heterogeneous cancer cell populations, a feature of high-grade lesions, also appear to be particularly primed for entosis induction, as differences in cell size can manifest as tension imbalances, and increased cell size is itself another feature that may favor the internalization of neighboring cells. Intriguingly, the process of entosis can further promote heterogeneity by disrupting cell division and generating multinucleated, grossly aneuploid cells^17^, in which differences in cell size can also manifest as a result of changes in ploidy. In this manner, entosis is both primed by heterogeneity and participates in feedforward signaling to promote this key feature of aggressive cancers. Entosis also allows winner cells to feed off of the loser cells that are internalized and killed. Several reports have shown that entotic hosts can utilize nutrients from digested cells to support their survival and proliferate when otherwise starved for exogenous nutrients^16,34^, a common condition in cancer tissues. Entosis can therefore select for cells with low-tension setpoints which is a hallmark of malignancy, promote the generation of grossly aneuploid cell lineages, and support cell lineages with large sources of scavenged nutrients.

Within mixed death responses, which may be more common in cell populations than currently appreciated, entosis may be one form of cell death that can drive cancer progression and should be avoided during therapeutic induction of cell death. Future studies characterizing potential mixed death responses during cancer therapy may shed further light on the frequencies at which entosis can be induced, as well as on mechanisms that can shift the balance from one form of death to another. A recent study linking cell-in-cell formation resembling entosis to the shielding of internalized cells from targeted immunotherapies may represent yet further evidence that entosis-like mechanisms involving the internalization of live cells should be inhibited to enhance the efficacy of cancer therapies^64^. While herein, we highlighted reports focusing on entosis and direct entosis-resembling processes to discuss their modes of regulation and roles in cancer, numerous additional forms of live-cell engulfment that can involve homotypic or heterotypic cell types have also been documented, for example, cell cannibalism^8^. Entotic-like mechanisms may also involve heterotypic cell types^65–68^, although herein, we focused primarily on homotypic engulfments. Processes of cell-in-cell formation are an emerging area of investigation, and understanding the roles of these various processes in complex diseases such as cancer may uncover new insights into the mechanisms that control disease progression and offer new insights into possible therapeutic interventions.
